# Supplementary material for: Genetic evidence for amlodipine’s protective role in gastroesophageal reflux disease: A focus on CACNB2
Source: PLoS One. 2025 Feb 18;20(2):e0309805. doi: 10.1371/journal.pone.0309805 (PMC11835245; doi:10.1371/journal.pone.0309805)
Supplement: S1 Checklist — (DOCX) [file pone.0309805.s001.docx]

**STROBE-MR checklist of recommended items to address in reports of Mendelian randomization studies**^1^ ^2^

| **Item No.** | **Section** | **Checklist item** | **Page No.** | **Relevant text from manuscript** |
| --- | --- | --- | --- | --- |
| 1 | **TITLE and ABSTRACT** | Indicate Mendelian randomization (MR) as the study’s design in the title and/or the abstract if that is a main purpose of the study | 1 | This study aims to elucidate the causal relationship between genetically predicted amlodipine use and the risk of gastroesophageal reflux disease (GERD) using a bidirectional Mendelian Randomization (MR) approach... |
|  | **INTRODUCTION** |  | 2 |  |
| 2 | **Background** | Explain the scientific background and rationale for the reported study. What is the exposure? Is a potential causal relationship between exposure and outcome plausible? Justify why MR is a helpful method to address the study question | 2 | The exposure of interest is amlodipine, which has been hypothesized to influence GERD based on previous observational studies. Mendelian randomization is employed to reduce bias and confounding, providing a more reliable estimate of the causal effect. |
| 3 | **Objectives** | State specific objectives clearly, including pre-specified causal hypotheses (if any). State that MR is a method that, under specific assumptions, intends to estimate causal effects | 2 | The primary objective of this study is to assess whether genetically predicted amlodipine use causally affects GERD risk using Mendelian randomization. |
|  | **METHODS** |  | 3 |  |
| 4 | **Study design and data sources** | Present key elements of the study design early in the article. Consider including a table listing sources of data for all phases of the study. For each data source contributing to the analysis, describe the following: | 3 |  |
|  | a) | Setting: Describe the study design and the underlying population, if possible. Describe the setting, locations, and relevant dates, including periods of recruitment, exposure, follow-up, and data collection, when available. | 3 | We conducted a bidirectional Mendelian Randomization (MR) study to explore the relationship between amlodipine and the risk of gastroesophageal reflux disease (GERD)... |
|  | b) | Participants: Give the eligibility criteria, and the sources and methods of selection of participants. Report the sample size, and whether any power or sample size calculations were carried out prior to the main analysis | 3 | Data on amlodipine and GERD were drawn both from the Neale Lab consortium... including 13693 cases and 323466 controls for amlodipine and 14316 cases and 322843 controls for GERD. |
|  | c) | Describe measurement, quality control and selection of genetic variants | 3 | Instrumental variable selection and data harmonization included SNPs that were genome-wide significant... |
|  | d) | For each exposure, outcome, and other relevant variables, describe methods of assessment and diagnostic criteria for diseases | 3 | The GWAS data used in our study were all publicly available and the participants were of European ancestry. |
|  | e) | Provide details of ethics committee approval and participant informed consent, if relevant | 3 | The data utilized in our analysis were openly accessible and had been authorized by the institutional review board in the relevant studies. |
| 5 | **Assumptions** | Explicitly state the three core IV assumptions for the main analysis (relevance, independence and exclusion restriction) as well assumptions for any additional or sensitivity analysis | 3 | We explicitly state the three core IV assumptions for the main analysis: relevance, independence, and exclusion restriction. Sensitivity analyses were conducted to assess pleiotropy and heterogeneity |
| 6 | **Statistical methods: main analysis** | Describe statistical methods and statistics used |  |  |
|  | a) | Describe how quantitative variables were handled in the analyses (i.e., scale, units, model) | 3 | For each instrument (SNP) we calculated the proportion of variance explained (R²) for both the exposure and the outcome... |
|  | b) | Describe how genetic variants were handled in the analyses and, if applicable, how their weights were selected | 3 | Genetic variants were selected based on linkage disequilibrium clustering and genome-wide significance. |
|  | c) | Describe the MR estimator (e.g. two-stage least squares, Wald ratio) and related statistics. Detail the included covariates and, in case of two-sample MR, whether the same covariate set was used for adjustment in the two samples | 4 | We used the Two-Sample MR package and methods including IVW, MR-Egger, and Weighted Median. |
|  | d) | Explain how missing data were addressed | 3 | Missing data were addressed by using proxy SNPs for linkage disequilibrium tagging. |
|  | e) | If applicable, indicate how multiple testing was addressed | 3 | Multiple testing was addressed by setting a threshold for genome-wide significance (P < 5 × 10−8). |
| 7 | **Assessment of assumptions** | Describe any methods or prior knowledge used to assess the assumptions or justify their validity | 3 | The MR-Egger intercept and MR-PRESSO methods were used to assess horizontal pleiotropy. Cochran’s Q statistic was used to assess heterogeneity." |
| 8 | **Sensitivity analyses and additional analyses** | Describe any sensitivity analyses or additional analyses performed (e.g. comparison of effect estimates from different approaches, independent replication, bias analytic techniques, validation of instruments, simulations) | 3 | Sensitivity analyses including leave-one-out analysis, MR-Egger intercept, and MR-PRESSO were performed to validate the robustness of the main results and to detect potential pleiotropy. |
| 9 | **Software and pre-registration** |  |  |  |
|  | a) | Name statistical software and package(s), including version and settings used | 4 | We performed MR analysis using R software (version 4.3.2) and the 'Two-Sample MR' package (version 0.5.11). |
|  | b) | State whether the study protocol and details were pre-registered (as well as when and where) |  | Not pre-registered |
|  | **RESULTS** |  |  |  |
| 10 | **Descriptive data** |  |  |  |
|  | a) | Report the numbers of individuals at each stage of included studies and reasons for exclusion. Consider use of a flow diagram | 5 | The analysis included 13693 cases and 323466 controls for amlodipine and 14316 cases and 322843 controls for GERD. Detailed reasons for exclusion and a flow diagram are provided in Supplementary Figure S1. |
|  | b) | Report summary statistics for phenotypic exposure(s), outcome(s), and other relevant variables (e.g. means, SDs, proportions) | 5 | Summary statistics for phenotypic exposures and outcomes, including means and SDs, are reported in Table 1 |
|  | c) | If the data sources include meta-analyses of previous studies, provide the assessments of heterogeneity across these studies | 5 | Assessment of heterogeneity across studies is provided in the supplementary materials. |
|  | d) | For two-sample MR:  i.  Provide justification of the similarity of the genetic variant-exposure associations between the exposure and outcome samples  ii.  Provide information on the number of individuals who overlap between the exposure and outcome studies | 3 | For two-sample MR, genetic variant-exposure associations were justified by the similarity in the exposure and outcome samples, with no overlapping individuals. |
| 11 | **Main results** |  |  |  |
|  | a) | Report the associations between genetic variant and exposure, and between genetic variant and outcome, preferably on an interpretable scale | 5 | Associations between genetic variant and exposure and between genetic variant and outcome are reported in Table 1. |
|  | b) | Report MR estimates of the relationship between exposure and outcome, and the measures of uncertainty from the MR analysis, on an interpretable scale, such as odds ratio or relative risk per SD difference | 5 | MR estimates show an inverse association between amlodipine and GERD risk (IVW OR = 0.872, 95% CI = 0.812-0.937, P = 0.0002). |
|  | c) | If relevant, consider translating estimates of relative risk into absolute risk for a meaningful time period | 5 | Relative risk estimates are provided; however, absolute risk estimates are not applicable in this context. |
|  | d) | Consider plots to visualize results (e.g. forest plot, scatterplot of associations between genetic variants and outcome versus between genetic variants and exposure) | 5 | Plots visualizing the results are provided in [Supplementary Figure S1-2](#_bookmark3) |
| 12 | **Assessment of assumptions** |  |  |  |
|  | a) | Report the assessment of the validity of the assumptions | 5 | The validity of the assumptions was assessed using MR-Egger intercept and Cochran’s Q test. No evidence of pleiotropy or heterogeneity was found. |
|  | b) | Report any additional statistics (e.g., assessments of heterogeneity across genetic variants, such as *I^2^*, Q statistic or E-value) | 5 | Additional statistics, including I2 and Q statistics, are reported in Table 1. |
| 13 | **Sensitivity analyses and additional analyses** |  |  |  |
|  | a) | Report any sensitivity analyses to assess the robustness of the main results to violations of the assumptions | 5 | Sensitivity analyses, including leave-one-out analysis, confirmed the robustness of the results. |
|  | b) | Report results from other sensitivity analyses or additional analyses | 5 | Additional sensitivity analyses using MR-PRESSO and robust adjusted profile score (MR.RAPS) were conducted. |
|  | c) | Report any assessment of direction of causal relationship (e.g., bidirectional MR) | 5 | Bidirectional MR showed no reverse causality for genetically predicted GERD and amlodipine. |
|  | d) | When relevant, report and compare with estimates from non-MR analyses |  | NA |
|  | e) | Consider additional plots to visualize results (e.g., leave-one-out analyses) |  | Additional plots, including leave-one-out analyses, are provided in Supplementary Figures S2. |
|  | **DISCUSSION** |  |  |  |
| 14 | **Key results** | Summarize key results with reference to study objectives | 6 | The MR analysis indicated a significant negative association between genetically predicted amlodipine use and GERD risk, with the IVW method showing an OR of 0.872 (95% CI 0.812-0.937, P = 0.0002) |
| 15 | **Limitations** | Discuss limitations of the study, taking into account the validity of the IV assumptions, other sources of potential bias, and imprecision. Discuss both direction and magnitude of any potential bias and any efforts to address them | 8 | The study relies on publicly available datasets, which may vary in quality and consistency. The lack of experimental validation is a limitation. Potential biases and imprecision are addressed through comprehensive sensitivity analyses. |
| 16 | **Interpretation** |  |  |  |
|  | a) | Meaning: Give a cautious overall interpretation of results in the context of their limitations and in comparison with other studies | 8 | Overall, the results suggest a protective effect of amlodipine against GERD, but should be interpreted cautiously due to reliance on secondary data. |
|  | b) | Mechanism: Discuss underlying biological mechanisms that could drive a potential causal relationship between the investigated exposure and the outcome, and whether the gene-environment equivalence assumption is reasonable. Use causal language carefully, clarifying that IV estimates may provide causal effects only under certain assumptions | 7 | The underlying biological mechanisms likely involve calcium ion transport and signaling pathways. The gene-environment equivalence assumption appears reasonable given the robust MR results. |
|  | c) | Clinical relevance: Discuss whether the results have clinical or public policy relevance, and to what extent they inform effect sizes of possible interventions | 7 | The findings suggest that amlodipine could be repurposed for GERD treatment, potentially informing clinical practice and public health strategies. |
| 17 | **Generalizability** | Discuss the generalizability of the study results (a) to other populations, (b) across other exposure periods/timings, and (c) across other levels of exposure | 8 | The generalizability of the results to other populations is supported by the use of European ancestry data. However, the applicability across different exposure periods and levels requires further investigation. |
|  | **OTHER INFORMATION** |  |  |  |
| 18 | **Funding** | Describe sources of funding and the role of funders in the present study and, if applicable, sources of funding for the databases and original study or studies on which the present study is based | 9 | This research received no specific grant from any funding agency in the public, commercial, or not-for-profit sectors. Summary statistics for the genetic associations were obtained from GWAS and GEO databases. |
| 19 | **Data and data sharing** | Provide the data used to perform all analyses or report where and how the data can be accessed, and reference these sources in the article. Provide the statistical code needed to reproduce the results in the article, or report whether the code is publicly accessible and if so, where | 9 | The original contributions presented in the study are included in the article/Supplementary Material. Further inquiries can be directed to the corresponding author. |
| 20 | **Conflicts of Interest** | All authors should declare all potential conflicts of interest | 9 | The authors declare that the research was conducted in the absence of any commercial or financial relationships that could be construed as a potential conflict of interest. |

This checklist is copyrighted by the Equator Network under the Creative Commons Attribution 3.0 Unported (CC BY 3.0) license.

1. Skrivankova VW, Richmond RC, Woolf BAR, Yarmolinsky J, Davies NM, Swanson SA, et al. Strengthening the Reporting of Observational Studies in Epidemiology using Mendelian Randomization (STROBE-MR) Statement. JAMA. 2021;under review.

2. Skrivankova VW, Richmond RC, Woolf BAR, Davies NM, Swanson SA, VanderWeele TJ, et al. Strengthening the Reporting of Observational Studies in Epidemiology using Mendelian Randomisation (STROBE-MR): Explanation and Elaboration. BMJ. 2021;375:n2233.
