# Supplementary material for: Genetic evidence for amlodipine’s protective role in gastroesophageal reflux disease: A focus on CACNB2
Source: PLoS One. 2025 Feb 18;20(2):e0309805. doi: 10.1371/journal.pone.0309805 (PMC11835245; doi:10.1371/journal.pone.0309805)
Supplement: Attachment — Submitted filename: Review report of PONE-D-24-22095 - [EMIDdce6b8afba788c30].docx [file pone.0309805.s006.docx]

**REVIEW REPORT OF PONE-D-24-22095 - [EMID: dce6b8afba788c30]**

Overall the **“Genetic Evidence for Amlodipine's Protective Role in Gastroesophageal Reflux Disease: A Focus on CACNB2”** is a very interesting research manuscript. Very much informative and well written, however needs some minor corrections.

1) The overall English is easy and understandable, however needs improvement.
2) Introduction chapter is relevant, however needs some latest inclusion of some references relevant to the context.
3) The methodology is relevant and very well written.
4) The results chapter is also written in a very good way.
5) Remove double spacing in **Study Design** line 3.
6) Check the last paragraph in **Study Design** for grammatical mistake.
7) Correct the spacing errors in **Data sources and processing** section.
8) Correct the grammatical mistake and spacing errors in section **Instrumental variable selection and data harmonization.**
9) No need to write Version as capital in section **Construction of ceRNA network of Drug Target Genes and Identification of Core Targets.**
10) Too many spacing errors. Kindly correct all the spacing errors in the overall research manuscript.

I accept the manuscript after the minor correction.
