# Supplementary material for: Genetic evidence for amlodipine’s protective role in gastroesophageal reflux disease: A focus on CACNB2
Source: PLoS One. 2025 Feb 18;20(2):e0309805. doi: 10.1371/journal.pone.0309805 (PMC11835245; doi:10.1371/journal.pone.0309805)
Supplement: Attachment — Submitted filename: comments for Plos one .docx [file pone.0309805.s007.docx]

**Review of "Genetic Evidence for Amlodipine's Protective Role in Gastroesophageal Reflux Disease: A Focus on CACNB2"**

**Major Concerns**

1. **Methodological Clarity and Justification**:
   - **Instrumental Variable Selection**: The paper describes the selection and clustering of SNPs but lacks detail on the criteria used to validate these SNPs as strong instruments. While the linkage disequilibrium and significance thresholds are mentioned, the justification for excluding certain SNPs is not clearly explained.
   - **Data Harmonization**: The harmonization process, especially handling palindromic and ambiguous SNPs, needs more clarity. The rationale behind proxy SNP usage and its impact on the results should be more thoroughly discussed.
2. **Results Interpretation**:
   - **Association vs. Causation**: The paper concludes a protective effect of amlodipine on GERD risk. However, the distinction between association and causation needs stronger emphasis, especially given the nature of MR studies.
3. **Functional Analysis and Biological Pathways**:
   - **Enrichment Analysis**: The connection between identified pathways and GERD is inferred but not deeply explored. How calcium ion transport and related pathways specifically influence GERD pathophysiology should be more explicitly discussed so that readers may better understand it.
4. **General**
   - **Comparison with Other Medications**: While amlodipine is highlighted, comparisons with other calcium channel blockers or antihypertensive medications are not well developed. This comparison could strengthen the argument for amlodipine's unique role.

**Minor Concerns**

1. **Writing and Presentation**:
   - **Abstract and Introduction**: The abstract is comprehensive, but the introduction could benefit from a clearer statement of the study's novelty and specific hypotheses.
   - **Figures and Tables**: The figures (e.g., funnel plots, enrichment analyses) are informative, but the legends should provide more contexts to aid interpretation.
2. **Statistical Methods**:
   - **Software and Packages**: While the use of R and specific MR packages is mentioned, a more detailed description of the statistical methods and assumptions would help readers replicate the study.
   - **Sensitivity Analyses**: The results of sensitivity analyses are presented, but the implications of findings like the MR-Egger intercept and Cochran’s Q-test are not explained.
3. **Technical Accuracy**:
   - **Genetic Terms and Definitions**: Some genetic terms (e.g., linkage disequilibrium, pleiotropy) are used without definitions. Including brief explanations would benefit readers who are not geneticists.
   - **Gene Lists and Databases**: The sources of gene lists and databases used for functional analysis are cited, but the process of selecting and validating these resources should be clearer.

**Decision: Minor revision**
