# Supplementary material for: Genetic evidence for amlodipine’s protective role in gastroesophageal reflux disease: A focus on CACNB2
Source: PLoS One. 2025 Feb 18;20(2):e0309805. doi: 10.1371/journal.pone.0309805 (PMC11835245; doi:10.1371/journal.pone.0309805)
Supplement: Attachment — Submitted filename: Response to Reviewers.docx [file pone.0309805.s008.docx]

**Review of "Genetic Evidence for Amlodipine's Protective Role in Gastroesophageal Reflux Disease: A Focus on CACNB2"**

**Major Concerns**

1. **Methodological Clarity and Justification**:
   - **Instrumental Variable Selection**: The paper describes the selection and clustering of SNPs but lacks detail on the criteria used to validate these SNPs as strong instruments. While the linkage disequilibrium and significance thresholds are mentioned, the justification for excluding certain SNPs is not clearly explained.
   - **Response:** Thank you for your insightful comment regarding the selection and clustering of SNPs. We agree that further clarification is needed. In our study, we selected SNPs based on genome-wide significance (P < 5 × 10^−8^) and ensured independence by clustering them with a linkage disequilibrium threshold of r² < 0.001 within a 10,000 kb window. This approach was chosen to minimize confounding due to linkage disequilibrium. SNPs were excluded if they did not meet these criteria or if they were identified as palindromic and ambiguous, as these can introduce biases in Mendelian Randomization (MR) analysis. We will revise the manuscript to provide a more detailed explanation of these criteria and their implementation. For example, we applied a PhenoScanner search to assess all known phenotypes related to the considered genetic instruments in our analyses.
   - **Data Harmonization**: The harmonization process, especially handling palindromic and ambiguous SNPs, needs more clarity. The rationale behind proxy SNP usage and its impact on the results should be more thoroughly discussed.
   - **Response:** We appreciate your concern regarding data harmonization. In our study, palindromic SNPs were carefully managed to avoid strand ambiguity, and ambiguous SNPs were excluded from the analysis to maintain data integrity. When necessary, proxy SNPs were used, selected based on high linkage disequilibrium (r² > 0.8) with the original SNPs to preserve the instrument's validity. We acknowledge that the impact of using proxy SNPs on the results can be significant and will include a discussion on the potential biases and limitations associated with this approach in the revised manuscript.
2. **Results Interpretation**:
   - **Association vs. Causation**: The paper concludes a protective effect of amlodipine on GERD risk. However, the distinction between association and causation needs stronger emphasis, especially given the nature of MR studies.
   - **Response:** We appreciate the reviewer's feedback on this critical point. While our Mendelian Randomization study suggests a potential causal relationship between amlodipine use and reduced GERD risk, we recognize that MR studies have inherent limitations and cannot establish causation definitively. We will revise the discussion section to emphasize the distinction between association and causation more clearly, acknowledging the assumptions and limitations of the MR approach, such as potential pleiotropy and unmeasured confounding.
3. **Functional Analysis and Biological Pathways**:
   - **Enrichment Analysis**: The connection between identified pathways and GERD is inferred but not deeply explored. How calcium ion transport and related pathways specifically influence GERD pathophysiology should be more explicitly discussed so that readers may better understand it.
   - **Response:** Thank you for pointing out the need for a deeper exploration of the identified pathways. Our enrichment analysis highlighted calcium ion transport and signalling pathways as key biological processes involved. These pathways are crucial in regulating smooth muscle tone, including the lower oesophageal sphincter (LES), which plays a significant role in GERD pathophysiology. We will expand the discussion to provide a more detailed explanation of how these pathways influence GERD development and the potential mechanisms through which amlodipine exerts its protective effects.
4. **General**
   - **Comparison with Other Medications**: While amlodipine is highlighted, comparisons with other calcium channel blockers or antihypertensive medications are not well developed. This comparison could strengthen the argument for amlodipine's unique role.
   - **Response:** We appreciate the reviewer’s suggestion regarding the comparison with other calcium channel blockers or antihypertensive medications. However, the scope of our study was specifically designed to focus on the potential protective effect of amlodipine against GERD. Due to the study’s design and the available data, we were not equipped to conduct a comprehensive comparison with other medications within this framework. Our study aimed to elucidate the genetic relationship between amlodipine use and GERD risk, utilizing a Mendelian Randomization approach. Expanding the analysis to include other medications would require additional data sets and potentially different methodological approaches, which were beyond the scope of the current study. We acknowledge that such comparisons could provide valuable insights and strengthen the argument for amlodipine's unique role. Therefore, we suggest that this could be an important direction for future research. Future studies that include direct comparisons between different classes of calcium channel blockers or antihypertensive drugs may further clarify the unique effects of amlodipine on GERD risk. Such research could help identify specific pharmacological properties of amlodipine that contribute to its protective effects, providing a more comprehensive understanding of its role in GERD management.

**Minor Concerns**

1. **Writing and Presentation**:
   - **Abstract and Introduction**: The abstract is comprehensive, but the introduction could benefit from a clearer statement of the study's novelty and specific hypotheses.
   - **Response:** Thank you for your constructive feedback. We agree that the introduction could better highlight the study's novelty and hypotheses. We will revise the introduction to explicitly state our research question and the novel aspects of our study, such as the application of a bidirectional MR approach and the construction of a ceRNA network to explore the genetic relationship between amlodipine use and GERD.
   - **Figures and Tables**: The figures (e.g., funnel plots, enrichment analyses) are informative, but the legends should provide more contexts to aid interpretation.
   - **Response:** We appreciate the feedback on the figures and tables. We will revise the figure legends to include more detailed explanations, ensuring that they provide sufficient context for readers to interpret the data accurately. This will include descriptions of the methods used, the significance of the results, and any relevant biological implications.
2. **Statistical Methods**:
   - **Software and Packages**: While the use of R and specific MR packages is mentioned, a more detailed description of the statistical methods and assumptions would help readers replicate the study.
   - **Response:** Thank you for highlighting the need for a more detailed description of our statistical methods. We will expand the methods section to include a comprehensive description of the statistical software, and packages used, as well as the assumptions underlying our MR analyses. This will help ensure that the study can be replicated by other researchers.
   - **Sensitivity Analyses**: The results of sensitivity analyses are presented, but the implications of findings like the MR-Egger intercept and Cochran’s Q-test are not explained.
   - **Response:** We acknowledge that the implications of our sensitivity analyses require further elaboration. The MR-Egger intercept and Cochran’s Q-test are crucial in assessing pleiotropy and heterogeneity, respectively. We will revise the manuscript to explain the significance of these tests and how they support the robustness and validity of our findings.
3. **Technical Accuracy**:
   - **Genetic Terms and Definitions**: Some genetic terms (e.g., linkage disequilibrium, pleiotropy) are used without definitions. Including brief explanations would benefit readers who are not geneticists.
   - **Response:** We appreciate the suggestion to provide definitions for genetic terms. We will include brief explanations of key genetic terms, such as linkage disequilibrium and pleiotropy, to enhance the manuscript's accessibility for readers from diverse backgrounds.
   - **Gene Lists and Databases**: The sources of gene lists and databases used for functional analysis are cited, but the process of selecting and validating these resources should be clearer.
   - **Response:** Thank you for pointing out the need for clarity in our selection and validation process for gene lists and databases. We will revise the manuscript to provide a detailed description of the criteria and methods used to select and validate these resources, ensuring transparency and reproducibility of our analysis.

**Decision: Minor revision**
